# Gendered ethnic choice effects at the transition to upper secondary education in Switzerland

**DOI:** 10.3389/fsoc.2023.1158071

**Published:** 2023-04-17

**Authors:** David Glauser, Rolf Becker

**Affiliations:** Department of Sociology of Education, Institute of Educational Science, University of Bern, Bern, Switzerland

**Keywords:** ethnic choice effects, gender, immigrant optimism, aspirations, educational inequalities, KHB decomposition, upper secondary education, Switzerland

## Abstract

Positive ethnic choice effects, namely a higher likelihood of attending more demanding educational tracks among students of immigrant origin compared to their native peers, are observed in many countries. Immigrant optimism, and thus the striving for upward social mobility, is seen as a key mechanism for explaining ethnic choice effects. However, research on this topic often ignores gendered educational pathways and trajectories. Based on data from German-speaking Switzerland on two school-leaver cohorts, our interest is on whether ethnic choice effects are observable for both female and male students whose parents were born in the Balkans, Turkey or Portugal. In addition, we examine the extent to which aspirations contribute to explaining ethnic choice effects for both genders. To disentangle the direct effect of a migration background and the mediating effect of aspirations on educational attainment at upper secondary level, we apply the reformulated KHB method in our analyzes. Overall, our findings indicate that migrant women have made up ground on their native peers between the two school-leaving cohorts, contributing to the widening of the gender gap within the migrant group of interest. Of particular importance, however, is our finding that ethnic choice effects are observed only for men, while we do not observe any evidence of ethnic choice effects in the sample of women. Consistent with previous findings, our results show that aspirations mediate part of the ethnic choice effect. Our findings support the consideration that the room for ethnic choice effects is related to the proportion of young men and women striving for academic education, with gender differences in this regard being particularly pronounced in education systems with a high degree of vocational specificity.

## 1. Introduction

Educational attainment is decisive for social integration with regard to various aspects such as income prospects, health, and access to welfare services over the life course (Kalter et al., 2018). Educational inequalities related to social origin or migration background can therefore have a long-lasting and detrimental effect on social integration (Heath et al., 2008; OECD, 2018). In the case of educational disparities that are attributable to an individual's migration background, research findings underline disadvantages for specific ethnic or migrant groups at different educational levels in many countries (Rözer and van de Werfhorst, 2017; Nauck, 2019). However, *positive ethnic choice effects*, namely a higher probability of attending more demanding educational tracks at upper secondary and tertiary level, are also reported for otherwise economically low-privileged ethnic groups when controlling for school performance and social origin (Heath and Cheung, 2007; Murdoch et al., 2016; Salikutluk, 2016; Dollmann, 2017; Tjaden, 2017; Gil-Hernandez and Gracia, 2018; Dollmann and Weißmann, 2020).

Positive ethnic choice effects are often explained by reference to aspirations for upward social mobility among students of migrant origin. This surplus of motivation is referred to as “immigrant optimism” (Kao and Tienda, 1995) and highlights that, given equal academic performance and social background, students with an immigrant background show higher levels of aspiration compared to their native peers. This is expected particularly for students whose parents have been socially relegated due to migration. In the case of Switzerland, positive ethnic choice effects have been reported by Griga (2014) and Tjaden and Scharenberg (2017) for youth whose parents migrated to Switzerland from the former Yugoslavia (due to the Yugoslav Wars), Turkey or Portugal (migrant workers).

However, where transition rates to academic and vocational tracks differ systematically by gender, we do not consider it meaningful to refer to ethnic choice effects for an ethnic group as a whole when, in fact, the variation between genders in regard to the outcome of interest is high. This variation is likely to have an impact on whether ethnic choice effects are observable for young women and men. This is particularly the case, the more gender-specific educational pathways within an education system are (Buchmann et al., 2008; Breen et al., 2010; Fleischmann and Kristen, 2014), which applies to education systems with a high degree of vocational specificity (Shavit and Müller, 2000).

Our aim in this paper is twofold. First, we examine whether gender differences in ethnic choice effects can be observed among migrant youth from the Balkans, Turkey or Portugal at the transition to upper secondary education. Second, we test whether aspirations contribute equally to explaining ethnic choice effects for both women and men. To answer our research questions, we apply the reformulated decomposition method proposed by Breen et al. (2018, known as the KHB method) to disentangle the extent to which ethnic choice effects on the decision to attend an academic track (gymnasium, specialized schools) instead of vocational education and training (VET) are mediated by aspirations for upward social mobility.

In our analysis, we use longitudinal data from two school-leaver cohorts: data from TREE, a follow-up to PISA 2000, covering the transition to upper secondary education of young people who left compulsory education in 2000; and data from the DAB panel study, which contain information on youth who left compulsory school in the summer of 2013. As the change in educational opportunities takes place through successive cohorts (Becker and Mayer, 2019; Blossfeld, 2020), the cohort comparison provides information on the extent to which the educational opportunities of women and men from the migration population of interest have changed over time and how these changes have contributed to a narrowing or widening of the gender gap. In addition, these two cohorts of school-leavers are considered because the data allow for the possibility that differences in the results between the two cohorts are may be due to differences in the time at which aspirations were measured. Although we are not interested in the development of aspirations in the course of the school career, in our view, this aspect is relevant because aspirations measured close to the actual transition may already be “cooled out” in the event of the failure to pass entrance examinations or to access apprenticeships (Heckhausen and Tomasik, 2002; Möser, 2022). This may lead to an underestimation of the mediating effect of aspirations when analyzing ethnic choice effects.

The remainder of this paper is structured as follows. After discussing why we expect gendered ethnic choice effects, we describe recent developments in immigration in Switzerland, provide a summary of educational pathways in the country, and then present our hypotheses. Having introduced the data and the analysis strategy, we present our results and conclude with a discussion.

## 2. Gendered ethnic choice effects— Theoretical background

Disadvantages in the educational attainment of youth with a migrant background are often attributed to *primary ethnic effects*, i.e., poorer academic performance than their majority peers at school entry and at later stages when controlling for social origin (Meunier, 2011; Borgna and Contini, 2014; Contini and Azzolini, 2016; Veerman and Dronkers, 2016; Spörlein and Schlueter, 2018; Kristen, 2019; Nauck, 2019; Becker and Klein, 2021). In contrast, however, advantages in educational trajectories— a higher propensity to opt for academic tracks at upper secondary and tertiary level— have also been observed for migrant groups in many countries when controlling for prior achievement and endowment with family resources (*secondary ethnic effects*; see Kilpi-Jakonen 2011; Tjaden and Scharenberg 2017; Hadjar and Scharf 2018; Dollmann and Weißmann 2020; Combet and Oesch 2021). The secondary ethnic effect refers to the paradoxical finding that, despite the educational disadvantages faced by young people from certain ethnic groups, migrants are more likely to opt for academic tracks than their native classmates, controlling for social class and prior school performance (Kristen et al., 2011; Salikutluk, 2016).

The ethnic choice effect is partly due to the high educational and occupational aspirations of young people with a migration background. High aspirations among migrant youth are often explained by three theoretical arguments, which are not mutually exclusive: immigrant optimism; anticipated discrimination; and information deficit (Heath et al., 2008; Relikowski et al., 2012; Tjaden and Hunkler, 2017; Neumeyer et al., 2022). *First*, familiarity with and knowledge of the education system and educational alternatives tend to be poorer in families with a migration background (Kristen and Olczyk, 2013; Forster and van de Werfhorst, 2020). This is more likely to be the case in countries like Switzerland, which is characterized by a historically developed and highly differentiated VET system. In this context, students whose parents have attained general education in the country of origin tend to overestimate the expected likelihood of attending and successfully completing general education (Relikowski, 2012).

*Second*, anticipated discrimination in the labor market may also reinforce high aspirations among minority youth because discrimination is less likely to occur in highly skilled jobs (Heath and Brinbaum, 2007; Jonsson and Rudolphi, 2011). Given that discrimination is anticipated, the expected returns from investing in education exceed the expected costs of discrimination. There is ample experimental evidence pointing to discrimination against ethnic minorities by employers (Switzerland: Zschirnt 2019, Netherlands and Germany: Thijssen et al. 2019, Denmark: Dahl and Krog 2018, for an overview see: Lancee 2019).

*Third*, and with reference to the *immigrant optimism hypothesis*, “[immigrant] parents” optimism about their socio-economic prospects leads youths to behave in ways that promote educational success' (Kao and Tienda, 1995, p. 5). The striving of migrant youth for upward social mobility—particularly in the case of economically motivated migration—is attributed to the fact that migrants are positively selected compared to non-migrants with regard to educational motivation and their aspiration for upward social mobility (Hadjar and Scharf, 2018; Spörlein and Kristen, 2019; Schmidt et al., 2022). Investing in education is therefore a promising opportunity for social mobility, even if the human capital acquired by parents in their country of origin is at best devalued by migration, which may hamper their ability to support the educational careers of their offspring (Nauck, 1994, 2001; Vallet, 2005). High aspirations are thus expected for the population of migrants irrespective of the social status of the family. In the majority population, however, children from socially privileged families, in particular, show a high educational motivation in order to avoid status loss (Breen and Goldthorpe, 1997; Kroneberg and Kalter, 2012), which is seen as a key mechanism for them being over-represented in more demanding tracks at different educational levels (Jackson, 2013; Blossfeld et al., 2016). Accordingly, differences in regards to the aspiration for upward social mobility between ethnic minority groups and the majority population are expected particularly in the lower tail of the social strata (Relikowski et al., 2009; Dollmann, 2017).

While we do not in any way question the underlying mechanisms that contribute to high educational and occupational aspirations among migrant youths, in our view, little attention has been paid so far to the context, namely how gendered educational trajectories are related to whether ethnic choice effects are observable for both genders and when they may not. Therefore, when turning to the *intersection of migration background and gender*, it should be emphasized that—as is the case within majority populations in Western countries—migrant girls usually outperform migrant boys in regard to school performance, as well as in their transition rates to programmes that qualify for higher education (Buchmann and DiPrete, 2006; Støren and Helland, 2010; Dronkers and Kornder, 2014; Fleischmann and Kristen, 2014; Blossfeld et al., 2015; SCCRE, 2018). Although higher educational aspirations are observed for migrant women than for migrant men (Feliciano and Rumbaut, 2005; Rampino and Taylor, 2013), and there are numerous studies of educational advantages for women over men, there is no evidence of educational advantages for migrant women over male migrants over and above ethnic selection effects (Jonsson and Rudolphi, 2011; Fleischmann and Kristen, 2014; Contini and Azzolini, 2016; Dollmann, 2017).

There are various reasons to assume that the ethnic choice effects observed for specific migration groups are not inevitably present for women and men. Gender gaps in regard to educational attainment within the majority population, as well as for migrant groups, depend on the overall proportion of students attending an academic track or VET at upper secondary level. Fleischmann and Kristen (2014) point out that gender differences become less likely, the higher the proportion of women and men attending academic tracks. The opposite can be expected the more gender-typed educational pathways are. This is particularly the case in education systems characterised by a high degree of vocational specificity (Müller and Shavit, 1998). In these education systems, the proportion of young men in general education is substantially lower compared to young women, whereas the opposite holds for VET (Imdorf et al., 2014; Kriesi and Imdorf, 2019; Leemann et al., 2022).

Since our focus is not on the gender gap *per se*, but on whether or not to expect gendered ethnic choice effects within the group of men and the group of women, respectively, we argue that where there are generally high educational and occupational aspirations within a population, the impact of aspirations on the decision to pursue an academic track is decreasing. The reason for this is seen in the fact that where a relatively high proportion of persons in a population acquire an academic track, the educational and occupational aspirations within this group are already pronounced. In education systems with a high degree of vocational specificity (Müller and Shavit, 1998), this is particularly the case for women, for whom the motivation to begin an academic track might be seen as a means to prevent them from being pushed into female-dominated, dead-end, and lower paying jobs after attaining a VET diploma (Buchmann and Charles, 1995; Estévez-Abe, 2006; Gundert and Mayer, 2012; Grønning et al., 2020). In such a context, a relatively high proportion of women aspires to enter occupations for which a higher education entrance qualification is required. In addition, these high educational aspirations are related to the tertiarisation of many women's occupations over recent decades (Buchmann et al., 2008; Kriesi and Imdorf, 2019; Basler et al., 2021; Becker and Blossfeld, 2021; Nießen et al., 2022; Wicht et al., 2022). If the proportion among women striving for academic education is relatively high, we assume that the room for ethnic choice effects is confined.^1^

This “ceiling effect” is assumed to fade out the lower the proportion of persons who aspire to an academic track. In countries with a highly differentiated VET system the proportion of students attending an academic track is comparatively low, and in Switzerland in particular in the population of young men, who are substantially more likely to opt for VET than women do (see section below and Kriesi and Imdorf, 2019). Apart from the influence of gendered educational trajectories, ethnic choice effects among male migrants might also be amplified by anticipated discrimination in the allocation of apprenticeships and on the labor market (Imdorf, 2017; Protsch and Solga, 2017; Zschirnt, 2019). Ethnic discrimination is particularly likely to occur in labor markets with a high proportion of small and medium-sized enterprises without professional recruiting structures, as is the case in Switzerland (Hunkler, 2014; Söhn, 2020). In order to circumvent discrimination by training companies, young immigrant men are more likely to opt for an academic track than their peers from the majority population when controlling for school performance. We assume that, overall, the aspects discussed in this section contribute to ethnic choice effects being more pronounced in the population of men.

## 3. Application to the Swiss context

Since the 1970s, a dual regime of migration and mobility has emerged through Switzerland in which facilitated entry, admission and stay are restricted to nationals of the EU/EFTA member states while much stricter admission rules are applied to third-country nationals (D'Amato et al., 2019). In the context of this contribution, it is relevant that, first, there was a sharp increase in immigration at the beginning of the 1990s due to the Balkan War; and that, second, migration has again increased significantly since the introduction of the free movement regime for EU/EFTA-nationals in 2002. Within the past 30 years, the proportion of the permanent foreign resident population has risen from 15% in 1990 to 25% in 2017 (OFS, 2018).

This development is mirrored by the proportion of pupils at lower secondary level belonging to various nationalities since 1990 (see Figure 2 in the Appendix).^2^ There was a steep increase of pupils from the Balkans, Turkey, or Portugal in the early 1990s from 4% to more than 10% in the year 2000. Since then, the proportion of pupils with these nationalities has remained rather constant. These ethnic groups achieve a significantly lower socio-economic status compared to the majority population (Gomensoro and Bolzman, 2019). In contrast, there has been a decline in the proportion of pupils of Italian or Spanish nationality, whose (grand)parents traditionally belonged to the migration group known as ‘guest workers'. Since 2002, however, the proportion of pupils from the neighboring countries of Germany, France, Austria and Liechtenstein has doubled to 4%. These changes also reflect the fact that the educational background of migrants has changed remarkably from primarily low skilled and blue-collar workers to highly skilled labor migrants, with 60% holding a tertiary degree since 2010 (Oesch, 2013; Wanner and Steiner, 2018; D'Amato et al., 2019).

In the Swiss education system, tracking into school types at *lower secondary level* takes place at the end of Grade 6. In some cantons, pre-gymnasium begins in Grade 7, while in the majority of cantons pre-gymnasium starts in Grade 9. Students, who themselves or whose parents were born in the Balkans, Turkey, or Portugal, are over-represented in school types with the lowest cognitive demands (basic requirements), and are under-represented in school types with advanced requirements, as well as in the pre-gymnasium (Beck, 2015; Glauser, 2015). At the transition to *upper secondary education* at the end of Grade 9, pupils must opt either for basic VET or an academic track (gymnasium, specialized schools). The set of educational alternatives is linked to the type of school attended during compulsory schooling. Based on institutional regulations, admission to academic tracks is granted to students who have attended a school type with advanced requirements or a pre-gymnasium, and have achieved a required grade point average or have passed an entrance examination. Students at academic tracks (gymnasium, specialized schools) earn a higher education entrance qualification, whereas students in the VET system acquire occupation-specific skills in one of about 240 fields, which mainly prepare individuals for labor market entry. The transition to upper secondary education is decisive for educational and occupational career prospects since the permeability between academic and vocational tracks is low (Fazekas and Field, 2013).

**Figure 1:**
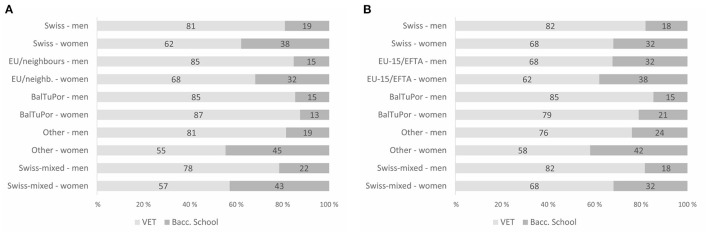
Upper secondary track attended, by migration background and gender. Remarks: Samples based on observations reported in Table 1. Estimates based on 25 imputed data sets using survey weights. Data: TREE/DAB; own calculations. **(A)** TREE sample. **(B)** DAB sample.

Although administrative data provide information only on students' nationality, we use these data for descriptive purposes to give an approximate overview of the distribution by nationality and gender at upper secondary level for the period between 1990 and 2015 (see Figures 3, 4 in the Appendix). The data highlight how gender-specific this educational trajectory is in Switzerland (see also Basler et al., 2021). The proportion of young Swiss men in VET is about 80%, but is higher for men with former Yugoslav, Turkish, or Portuguese nationality. In contrast, the proportion of Swiss women in VET is roughly two thirds while the proportion in academic tracks is higher than 25%, which is about ten percentage points higher than the figure for Swiss men. Overall, the gender differences are similar in the group of Swiss students and the migration group of interest, although the gender gap is larger in the latter group (Laganà et al., 2014).^3^ However, while the administrative data considered here are suitable for depicting gender differences in educational attainment, substantial differences are likely to emerge depending on whether the nationality of the students or the country of birth of the parents is used to operationalise the migration background of the students (Gresch and Kristen, 2011). Based on the survey data used in our empirical analyzes (see Figure 1 in Section 5), we conclude that young women in the migration group of interest have made up ground remarkably on women in the majority population. This change is paralleled by an increase in the gender gap between the 2000 and 2012 school-leaving cohorts for the former.

On the basis of the recent developments in migration to Switzerland described above, and previous findings by Tjaden and Scharenberg (2017) as well as Griga (2014), we expect ethnic choice effects among students from the Balkans, Turkey, or Portugal in terms of a higher propensity to be enrolled in academic tracks compared to their peers from the majority population. However, with reference to our theoretical argumentation, we assume there to be gender differences with regard to the observable ethnic choice effects (*Hypothesis 1*). Since women on average show a markedly higher transition rate to academic tracks than men, the room for ethnic choice effects within the population of women is likely to be limited. Given that female students from the ethnic groups of interest have made up ground considerably on young women from the majority population in terms of educational attainment within the period under examination, we expect to observe an ethnic choice effect within the older school-leaver cohort, but at most weak effects for female migrants within the younger cohort (*Hypothesis 2*). For men, however, the relative proportion enrolled in academic tracks is remarkably low and did not change substantially between the two cohorts. In this context, we expect the striving for upward social mobility to result in positive ethnic choice effects for men in both cohorts *(Hypothesis 3)*. Finally, we assume that part of the ethnic choice effects—if observable—is mediated by students' aspirations for upward social mobility *(Hypothesis 4)*.

## 4. Data, variables and analysis strategy

### 4.1. Data and sample

Our analysis is based on data from two school-leaver surveys. We use data from the first TREE cohort, which is a nation-wide Swiss panel study of young people born around 1985 who participated in PISA 2000 and left compulsory school in the same year (TREE, University of Bern, 2016). The analysis sample is restricted to respondents from German-speaking Switzerland in the second wave collected in 2002. By this time, the majority of the cohort had started education or training at upper secondary level. Additionally, recent data from the DAB panel study are used (DAB et al., 2022). The sample covers the transition to upper secondary education within German-speaking Switzerland for the cohort born around 1997 who left compulsory school in summer 2013. Information on the attended educational track at upper secondary level is based on the fourth wave of DAB, collected approximately 15 months after pupils had left compulsory school.

We consider only students with observed information on our main dependent variable: the upper secondary track attended (*N*_TREE_ men/women: = 1044/1233; *N*_DAB_ men/women: = 1054/1084). A small number of youth who were attending non-mandatory bridge year courses at the time of the survey are excluded (TREE: 214; DAB: 98). We impute missing information through chained equations (White et al., 2011) to generate 25 complete data sets. The imputation model includes several auxiliary variables, in addition to our analysis variables.^4^

### 4.2. Variables

Our main dependent variable is the upper secondary track attended. In order to test for ethnic choice effects, we distinguish between whether young people attend a vocational or academic track (gymnasium or specialized school) 15 months (DAB) to 2 years (TREE) after leaving compulsory school.

Regarding the underlying mechanism of the ethnic choice effect, and in line with status position theory (Keller and Zavalloni, 1964), we operationalise the aspiration for upward social mobility—our mediator variable—as the difference in status between the desired occupation of the child and the actual occupation of the parents.^5^ Desired and parental occupations were coded using the Swiss Standard Classification of Occupations and then converted into the International Standard Classification of Occupations (ISCO-08). The social status of the desired and parental occupations was then operationalised using the International Socio-Economic Index of Occupational Status (ISEI-08, Ganzeboom et al., 1992). The variable takes on positive values if the ISEI of the students' occupational aspiration is higher than the highest ISEI of the actual occupation of the parents.^6^ In previous research, students' desired occupation has usually been used to operationalise aspirations.^7^

Both data sets contain information on the country of birth of the parents and the youth. Our main interest is to compare students whose parents were born in the Balkans, Turkey or Portugal with students whose parents were born in Switzerland.^8^ Additionally, we differentiate whether both parents were born in EU/EFTA member states or in other countries, and whether each of the parents was born in a different country (i.e., Swiss/EU or EFTA, Swiss/other). To account for generation status, the students' country of birth is used as a control. Given our main research questions and since migration to Switzerland is polarized between highly and low skilled labor migrants (Oesch, 2013), our interest is on ethnic choice effects of first- and second-generation pupils whose parents were born in the Balkans, Turkey or Portugal.^9^

We measure the social origin of youth using the highest educational level (ISCED-97: tertiary education = 4/6 vs. non-tertiary = 1/3) and the highest socio-economic status (ISEI-08) within the family. To account for prior achievement we consider students with observed information on the school type attended from Grade 8 in the case of the DAB sample, while the TREE sample includes students from Grade 9. The grade point average (GPA) in mathematics and language (German) are used to control for school performance. We differentiate between the school type with basic requirements, the school type with advanced requirements, and the pre-gymnasium. Grades in Switzerland are usually ranked from 1 to 6, with 6 being the highest possible grade and 4 the minimum requirement to pass. Grades are used in *z*-standardised form. Descriptives of the variables used in our analysis are provided in the Appendix (see Tables 3, 4 in the Appendix).

### 4.3. Analytical strategy

While stratifying by school-leaver cohort and gender, the focus is on ethnic choice effects regarding enrolment in academic tracks (gymnasium or specialized schools). As proposed by Breen et al. (2018), we use the reformulated KHB method to predict rescaled logit coefficients of nested models, adapting the linear predictor method. In this approach, the binary dependent variable is regressed on all *X* variables, including the mediator variable *Z* (in our case, the aspirations of youth), using binary logistic regression. Based on this model, the latent index *xb* is saved and used as a new dependent variable using ordinary least squares (OLS), while covariates and mediators are included stepwise to estimate the extent of mediation. We present bootstrapped standard errors of selected coefficients of nested models, as well as estimates and bootstrapped standard errors of the indirect effects and the percentage mediated.^10^ Since we use imputed data, we follow the routine proposed by Little and Rubin (2002, p. 87) to calculate the bootstrap standard errors. Although this calculation is highly time-consuming and could be reduced when using a smaller number of imputed datasets (Schomaker and Heumann, 2018; Brand et al., 2019), we use 25 imputed datasets in both steps of our analysis, while we use 500 replications to calculate the bootstrap standard errors. We conduct the analyzes in both cohorts separately for women and men.

## 5. Results

Before turning to the multivariate analysis of the ethnic choice effects, we briefly outline some descriptives for the two cohorts (see Figure 1). In line with the administrative data presented above, the TREE and DAB data point to a substantial gender gap in the attended educational track at upper secondary level. Whereas more than 30% of women from the majority population start upper secondary education at a gymnasium or a specialized school (TREE: 38%; DAB: 32%), the proportion of men doing so in the majority population is remarkably low (TREE: 19%; DAB: 18%). Concerning the migration group of interest, the descriptives indicate no changes for men over time (TREE/DAB: 15%), while the proportion of women in academic tracks has increased significantly from 13% in the older cohort (TREE) to 21% in the younger cohort (DAB).^11^ Young women whose parents were born in the Balkans, Turkey or Portugal have thus made up ground up on their peers from the majority population, and they attend an academic track in a significantly higher proportion than young men with the same migration background do.

Next, we discuss our findings related to gendered ethnic choice effects. Table 1 shows the estimated association, using the linear predictor method, between migration background, as well as other control variables, and the chance that respondents in the TREE and DAB sample attend an academic track (gymnasium or specialized schools) instead of VET. Our main interest is the direct effects for youth whose parents were born in the Balkans, Turkey or Portugal, compared to their peers from the majority population. In Model 1 we control only for the migration background of the students. An insignificant association is observed for male students from the ethnic groups of interest compared to their peers from the majority population. In contrast, the results for female students indicate that women from these ethnic groups show a significantly lower propensity to enter an academic track than women whose parents were born in Switzerland.

**Table 1:** Educational situation two years (TREE)/15 months (DAB) after leaving compulsory education (0 = VET; 1 = academic track).

	**TREE men**			**TREE women**		
	**1**	**2**	**3**	**1**	**2**	**3**
EU/neighb. countries (*Ref*.: Switz.)	−1.377	0.177	−0.045	−0.270	0.109	−0.076
Balkans, Turkey, Portugal	−0.603	2.427	2.219	−2.099	−0.497	−0.645
	(0.771)	(0.664)	(0.646)	(0.605)	(0.532)	(0.522)
Other	−1.060	−0.080	−0.121	0.504	0.163	0.057
Swiss-mixed	0.240	0.319	0.288	0.383	0.209	0.056
Born abroad (*Ref*.: Switz.)	0.730	0.334	0.302	−0.314	0.043	0.029
Parental HISEI		0.027	0.053		0.015	0.035
ISCED 4-6 (*Ref*.: ISCED 1-3)		0.749	0.712		0.501	0.471
Basic requirements (*Ref*.: adv.)		−3.509	−3.176		−2.198	−2.038
Pre-gymnasium		3.292	2.866		2.457	2.128
GPA language		0.351	0.327		0.106	0.085
GPA mathematics		0.191	0.153		0.231	0.201
Aspirations			0.031			0.024
Constant	−3.372	−4.936	−6.174	−0.826	−2.267	−3.133
Observations	1044			1233		
# Bootstrap replications	487			498		
	**DAB men**			**DAB women**		
	**1**	**2**	**3**	**1**	**2**	**3**
EU15/EFTA (*Ref*.: Switz.)	1.349	1.870	1.638	0.784	1.242	1.293
Balkans, Turkey, Portugal	−0.194	1.579	0.905	−1.398	0.496	0.221
	(0.479)	(0.417)	(0.392)	(0.452)	(0.287)	(0.257)
Other	0.808	1.243	0.635	0.866	1.373	1.106
Swiss-mixed	0.254	0.738	0.407	0.073	0.388	0.267
Born abroad (*Ref*.: Switz.)	−0.350	−0.080	−0.247	−1.249	−0.749	−0.776
Parental HISEI		0.020	0.070		0.021	0.057
ISCED 4-6 (*Ref*.: 1-3)		0.538	0.255		0.488	0.261
Basic requirements (*Ref*.: adv.)		−1.769	−1.076		−4.469	−3.958
Pre-gymnasium		2.992	2.436		2.465	2.053
GPA language		0.760	0.666		0.540	0.460
GPA mathematics		0.194	0.162		0.370	0.291
Aspirations			0.052			0.038
Constant	−2.705	−3.953	−6.275	−1.695	−2.798	−4.696
Observations	1054			1084		
# Bootstrap replications	498			442		

Logit coefficient measured on the scale of the full model and selected bootstrapped SEs in parentheses. Estimates based on 25 imputed data sets using survey weights. Data: TREE/DAB; own calculations.

In Model 2, we additionally control for social origin, the school track attended at lower secondary level and school performance. As expected, we find gender differences in regard to the observable ethnic choice effects (*Hypothesis 1*). More concretely, our results reveal ethnic choice effects in the case of men in both cohorts. The positive association of young men from the ethnic groups of interest with the propensity to enter an academic track is stronger in the older cohort (TREE). This is in stark contrast to the findings for women for whom ethnic choice effects are not evident at all. Although the coefficient for women in the DAB sample is positive, albeit not significant, the coefficient for women in the TREE sample is negative even after controlling for social origin and the indicators of school type and school performance. While we expected this result for men (*Hypothesis 3*), we had also assumed that an ethnic choice effect for women in the older cohort would be present (*Hypothesis 2*).

Overall, these findings support our assumption that the room for ethnic choice effects is related to the relative proportion among men and women striving for academic education. The higher this proportion is within a population, the less room there is for ethnic choice effects. In Switzerland, this applies to the population of women, in which we do not observe ethnic choice effects for the ethnic groups of interest. Furthermore, it is important to note that had we conducted the same analyzes without stratifying by gender, we would have observed ethnic choice effects in both samples, although marginally above the level of *p* < .05 in the TREE sample (see Supplementary Table S8).

In Model 3, we additionally include the mediator variable: the aspiration for upward social mobility. Since we do not observe ethnic choice effects in the case of women, we discuss only the results of the mediation analysis for men. The estimates of the indirect effects and percentage mediated by aspirations are provided in Table 2. Accounting for aspirations leads to a decrease in the direct effect of migration background for the ethnic group of interest, although the decline is obviously larger in the younger cohort. The indirect effects are statistically significant for both cohorts, meaning that part of the observed ethnic choice effects can be attributed to the high aspirations among men of the migration group of interest, which is in line with our expectations (*Hypothesis 4*).

**Table 2:** Selected indirect effects and percentage mediated of migration background on upper secondary track attended in Table 1.

	**TREE**		**DAB**	
	**Men**	**Women**	**Men**	**Women**
Indirect effects				
M2 ⇒ M3	0.200 (0.086)	–	0.621 (0.137)	–
Percent mediated				
M2 ⇒ M3	8.4% (4.9)	–	40.1% (16.5)	–
# Bootstrap replications	491	–	499	–

Logit coefficient, percentage mediated and bootstrapped SEs in parentheses.

Estimates based on coefficients reported in Table 1.

While aspirations reduce the direct effect of the migration background on the propensity to attend an academic track by 8% in the older cohort (TREE), about 40% of the direct effect is mediated by aspirations in the younger cohort (DAB). However, the apparently low contribution of aspirations to the explanation of the ethnic choice effect in the TREE sample cannot be attributed to the fact that aspirations in this sample were measured only a few weeks before the end of compulsory schooling. Although it is theoretically plausible to assume that experiences in the form of missed entrance exams lead to a cooling-out of career goals and aspirations (Clark, 1960; Heckhausen and Tomasik, 2002; Neumeyer et al., 2022), i.e., an adjustment of the desired occupational career and the potentially still realizable training options leading to this goal. Accordingly, the mediating effect of aspirations may be underestimated when aspirations are measured close to the actual transition. However, our robustness checks with data from the DAB sample provide no evidence for such a relationship. If we consider in our analysis information on the school type attended, school performance and professional aspirations measured 2 months before the end of the 9th and final school year (see Supplementary Tables S9, S10), the indirect effects and the percentage of the direct effect that is mediated by aspirations hardly differ. Unfortunately, the TREE data do not allow us to determine whether the results would have been different if aspirations had been measured earlier. Whether our conclusion based on the data of the DAB sample can be extended to the TREE sample cannot be conclusively answered. Nevertheless, our main conclusion does not differ between the two school-leaving cohorts: we observe ethnic choice effects only among the young men, but not among the young women of the ethnic groups considered in the analyzes.

## 6. Discussion and conclusion

In the present study, our focus is on the presence of ethnic choice effects in German-speaking Switzerland among young men and women whose parents were born in the Balkans, Turkey or Portugal. These ethnic groups achieve a significantly lower socio-economic status compared to the majority population (Gomensoro and Bolzman, 2019), and for their children not only the strongest disadvantages in the education system but also ethnic choice effects have been reported (Griga, 2014; Tjaden and Scharenberg, 2017). In order to analyze the magnitude of ethnic choice effects at the trajectory to academic and vocational tracks at upper secondary level by gender, we use data on two school-leaver cohorts from 2000 (TREE) and 2013 (DAB). Besides addressing gendered ethnic choice effects, we analyze the extent to which ethnic choice effects in attending an academic track (gymnasium or specialized schools) instead of vocational training are mediated by aspirations. In order to disentangle the direct and indirect effects of migrant background on enrolment in upper secondary education, we applied the reformulated KHB method (Breen et al., 2018).

As an extension to the current state of research, we operationalise the aspiration for upward social mobility as the difference between the socio-economic status of the family and the desired occupation of the child. In this way, and in line with status position theory (Keller and Zavalloni, 1964), we aim to capture the aspiration for upward social mobility as the relative distance between ambition and social class. With reference to the immigrant optimism hypothesis (Kao and Tienda, 1995), we provide evidence of how aspirations for upward social mobility differ between ethnic minority groups and the majority population. In line with our theoretical reasoning and previous research, we observe the highest aspirations among youth within the migrant group of interest here, and particularly in the lower tail of the social strata. However, our results underline that differences in aspirations are stronger among men. These differences are less pronounced within the population of women. In our view, the observed gender differences are related to the tertiarisation of women's occupations and the higher proportion of women pursuing academic careers, which is associated with higher aspirations on average among women.

Our contribution highlights that ethnic choice effects for specific migration groups are not necessarily observed for both men and women. While existing research reveal ethnic choice effects at different educational levels, our findings underline the importance of analyzing ethnic choice effects separately by gender. This should be all the more important, the more gender-specific the educational pathways within an education system are. Our results indicate that the room for ethnic choice effects is related to the relative proportion among men and women striving for academic education. We expect similar results in other education systems characterized by a high degree of vocational specificity, as these systems often feature a high degree of gender segregation at post-compulsory level. In such a context, we do not consider it meaningful to refer to ethnic choice effects for an ethnic group as a whole when, in fact, the variation between genders in regard to the outcome of interest is high.

Our findings reveal the importance of conducting analyzes related to ethnic choice effects stratified by gender. We observe ethnic choice effects in both cohorts in the case of men, whereas such effects are not evident in the case of women. If we had conducted the analyzes without stratifying by gender, we would have observed ethnic choice effects in both samples, which is in line with previous findings (Tjaden and Scharenberg, 2017). However, we do not conclude that ethnic choice effects *per se* differ in other education systems by gender. Nevertheless, we strongly encourage scholars to replicate our findings with reference to migration groups in other countries for which gender-typical educational trajectories are characteristic.

With regard to the gendered ethnic choice effects reported here, we would like to emphasize that our results do not imply that young women from the migration group of interest are less successful in the education system than their male peers. On the contrary, young women from this migration group were able to catch up substantially with women of the majority population within the period considered. This has contributed to the widening of the gender gap within the migrant group of interest.

Finally, in terms of the limitations of our study, we faced the problem of many studies comparing majority and minority ethnic groups: The sample size of minority students limits the possibilities for analysis. In the context of tracked education systems such as that in Switzerland, it would be beneficial to further restrict the analysis sample to students from the cognitively more demanding school types at lower secondary level, who show a higher transition rate to an academic track than students from the school type with basic requirements. However, this was not possible due to sample size limitations as is the case for separate analyzes by ethnic origin. Although we have referred to the concept of immigrant optimism and used an operationalisation that captures aspirations for upward social mobility, it is clear that we cannot compare the aspirations of young people from the migrant group of interest here with those from the same country of origin who have not migrated. While we contribute to research on ethnic choice effects, further research should also consider additional mechanisms, such as anticipated or experienced discrimination, that may contribute to the explanation of ethnic choice effects.

## Data availability statement

Publicly available datasets were analyzed in this study. This data can be found at: DAB: https://doi.org/10.48573/dqgk-ja58; Repository: www.swissubase.ch; Ref. No. of data set: 946; TREE: https://doi.org/10.23662/FORS-DS-816-7; Repository: www.swissubase.ch; Ref. No. of data set: 816.

## Author contributions

DG performed the statistical analysis and wrote the first draft of the manuscript. All authors contributed to manuscript revision, read, and approved the submitted version.

## Footnotes

^1^We do not rule out that female aspirations might be mitigated or reinforced by traditional gender roles (Fleischmann and Kristen, 2014), parents' attitudes to liberal values (Nauck, 2001) or other aspects (Feliciano and Rumbaut, 2005; Kroneberg et al., 2021), but we expect a high variation in this respect within the migration group of interest here, which is why we classify these aspects as of lower importance.

^2^Swiss administrative data only provide information on the nationality of the students, while information on the country of birth of the parents and their child is not available. The proportions of students with a migration background are likely to be underestimated if only the nationality of the students is considered (Gresch and Kristen, 2011).

^3^There are substantial differences in the proportion of students in VET and academic tracks between different language regions. The proportion of students attending VET is highest within German-speaking Switzerland, while the proportion of students in academic tracks is considerably higher in French-speaking cantons [Federal Statistical Office (FSO), 2018]. The data we use in our analyzes refer only to young adults in German-speaking Switzerland.

^4^The following auxiliary variables are used in the imputation model for the DAB sample: The stratum of the DAB sampling reflecting combined information with regards to the commune type, school type, and proportion of migrants within a school; the commune type, a class level identifier from the sampling, gender, the attended school type in grade 9, the attended upper secondary track 15 months after leaving compulsory education. For the TREE sample we use the PISA 2000 sampling stratum, a class level identifier from the sampling, gender, the commune type, the attended school type in grade 9, and the attended upper secondary track 2 years after leaving compulsory education as auxiliary variables in the imputation model.

^5^In the TREE sample, students were asked in PISA 2000 “What kind of job do you expect to have when you are about 30 years old?”. In the DAB sample, students were asked “When you think about your future, what do you think your future profession will be?”.

^6^Descriptives of the generated indicator for aspirations are provided in Tables 3, 4 in the Appendix. There are differences between the samples in terms of the mean (Mean TREE men/women: 1.9/1.3; DAB men/women: -3.1/3.4), but the variance is greater in the DAB sample (SD TREE men/women: 19.6/19.2; SD DAB men/women: 27.3/26.1). Analyzes of differences in aspirations by migrant status, school type and gender are provided in the Supplementary material (see Figure S5 and Table S5).

^7^Results are only marginally different when the students career aspiration is used instead of the aspiration for upward social mobility. We report results of analyzes in which the career aspiration is used as a proxy for aspirations (see Supplementary Tables S6, S7).

^8^Within the former group of students, about 78% of the parents were born in the Balkans, 14% in Turkey, and 8% in Portugal.

^9^Due to the limited sample size, it is not possible to distinguish between the aforementioned ethnic groups and a more fine grained version of the generational status, e.g., first-, second-, and 2.5-generation (Kristen and Dollmann, 2010).

^10^Coefficients are considered significant at least at the 5% level when the ratio of point estimate and standard error is higher than the absolute value of 2. We refrain from using asterisks to indicate significant coefficients.

^11^Overall, in the TREE sample, the proportion of young women in an academic track is around 37% compared to 19% of young men. In the DAB sample, the proportion of young women in an academic track is 31%, compared to 13% of young men (see Tables 3, 4 in Appendix).
