# Supplementary material for: Gendered ethnic choice effects at the transition to upper secondary education in Switzerland
Source: Front Sociol. 2023 Apr 17;8:1158071. doi: 10.3389/fsoc.2023.1158071 (PMC10150114; doi:10.3389/fsoc.2023.1158071)
Supplement: Supplementary file 1 [file Data_Sheet_1.pdf]

frontiers

Supplementary Material

Comment on Figure 5: Even though the variation in aspirations between individuals of different ethnic groups is not the main focus of our analyses, we empirically test the assumption that differences in regards to the aspiration for upward social mobility between ethnic minority groups and the majority population are expected particularly in the lower tail of the social strata. For this purpose, and stratified by school-leaver cohort and gender, we provide results in regards to whether youth whose parents were born in the Balkans,

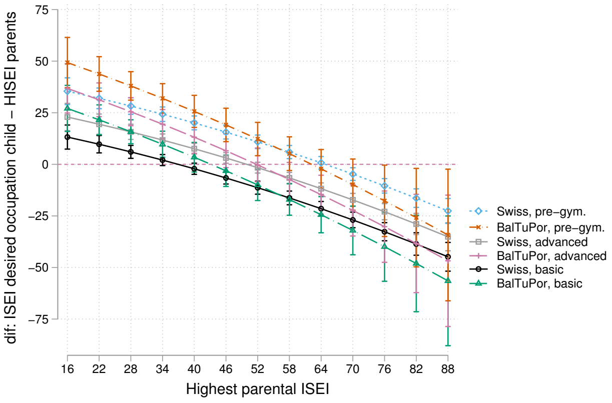

Figure 5a. TREE sample – men

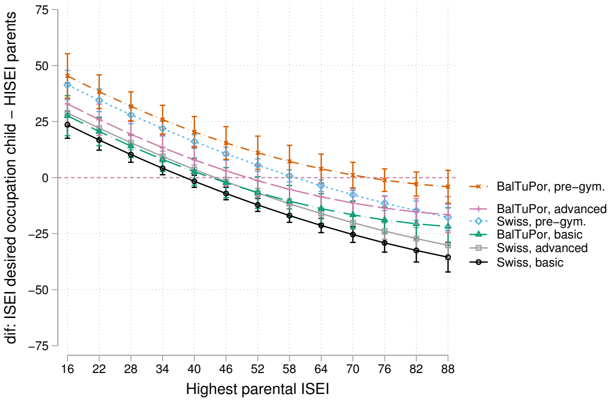

Figure 5b. TREE sample – women

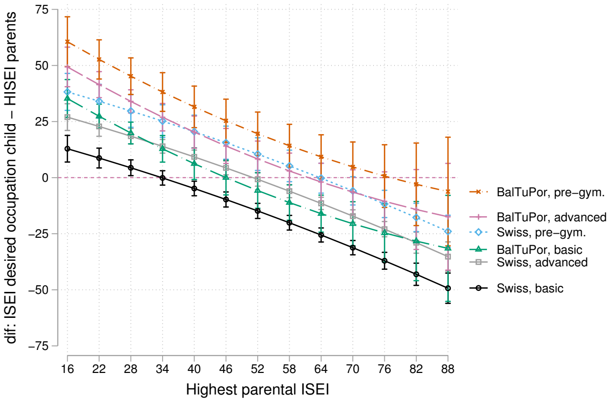

3

Figure 5c. DAB sample – men

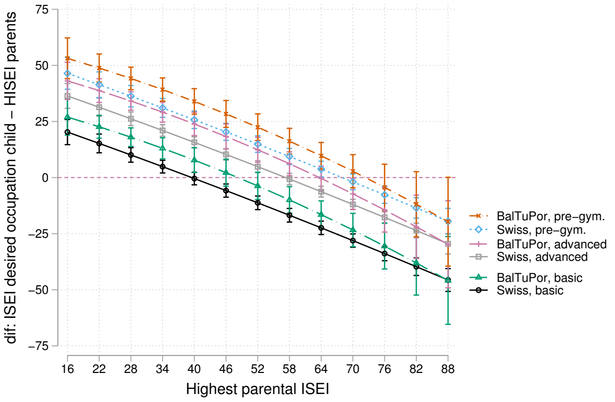

Figure 5d. DAB sample – women

Remarks: Plots based on extended models of Table S5 in which an interaction term for highest parental ISEI and ethnic background is included. To allow for a non-linear function between ISEI and ethnic background, we consider the ISEI in squared form in the three-way interaction. In Figure 5, the predictions of the status difference between a students' desired occupation and the highest parental ISEI (Y-axis) are plotted against the parental ISEI by the school type attended at lower secondary level. Predictions above the value 0 (red dotted line) indicate that youth aspire to upward social mobility. Plots based on 25 imputed data sets using survey weights. *Data*: TREE/DAB; own calculations

Figure 5. Predictive margins of the difference between the ISEI of the students' desired occupation and the highest ISEI of the parents (three-way interactions: highest ISEI of the parents × parental HISEI^2 × ethnic background)

Turkey or Portugal show higher aspirations for upward social mobility compared to the majority population using linear regression models (see Table S5). Overall, our findings reveal significant differences in aspirations for upward social mobility between youth whose parents were born in the Balkans, Turkey or Portugal, and those from the majority population. If we only consider the ethnic background, the highest aspiration for upward mobility is observed among youth from the mentioned ethnic groups (see Models 1, 3, 5 and 7). There are no substantial differences between the two cohorts in this regard, but the coefficients are stronger in the case of the younger cohort and for men. When we additionally control for social origin, the school type attended at lower secondary level and grades in language and mathematics, we find a similar pattern (see Models 2, 4, 6 and 8). Although the relevant coefficients are smaller compared to the reduced models, we still observe significantly higher aspirations among youths whose parents were born in the Balkans, Turkey or Portugal.

To obtain a more differentiated picture, we also estimate models in which an interaction term for the highest parental ISEI and ethnic background is included. Since we do not assume a linear function between ISEI and ethnic background, we consider the ISEI in squared form in the 3-way interaction, in order to account for nonlinearities. The results are provided graphically (see Figure 5). The predictions of the status difference between a students' desired occupation and the highest parental ISEI (Y-axis) are plotted against the parental ISEI by the school type attended at lower secondary level. Predictions above the value 0 (red dotted line) indicate that youth aspire to upward social mobility. In both cohorts, and for students in each of the three different school types, we observe higher aspirations among youth whose parents were born in the Balkans, Turkey or Portugal, compared to those from the majority population. While most of the confidence intervals in the older cohort overlap, the differences in aspirations are more pronounced in the younger cohort, but particularly for young men (Figure 5c). In line with theoretical expectations derived from the immigrant optimism hypothesis (Kao and Tienda, 1995), and previous findings by Dollmann (2017), the largest differences between youth from the ethnic groups of interest and those from the majority population are observed in the lower tail of the parental ISEI distribution, and fade out the higher the socio-economic status of the parents. This pattern is more apparent in the younger cohort (DAB).

	TREE men		TREE women		DAB men		DAB women	
	1	2	3	4	5	6	7	8
EU/neighbours (*Ref.*: Switz.)	4.360	6.005*	9.724*	7.381*	6.135	5.156	−2.566	−0.364
	(4.332)	(2.805)	(4.601)	(3.153)	(5.921)	(4.629)	(4.268)	(3.372)
Balkans, Turkey, Portugal	13.796***	7.717**	15.797***	5.447*	22.708***	12.718***	16.632***	6.391**
	(3.558)	(2.853)	(3.013)	(2.130)	(2.796)	(2.275)	(3.568)	(1.940)
Other	0.171	−1.173	1.396	4.766	16.192***	13.314***	13.718**	6.652**
	(3.379)	(3.049)	(6.462)	(3.678)	(4.266)	(2.575)	(4.804)	(2.205)
Swiss-mixed	−1.100	1.196	2.350	3.769*	3.673	6.910**	−0.706	3.113*
	(2.539)	(2.193)	(1.878)	(1.192)	(2.887)	(2.160)	(2.127)	(1.450)
Born abroad (*Ref.*: Switz.)	1.773	1.153	−2.137	−1.085	−2.761	0.570	−6.752*	1.040
	(3.303)	(2.591)	(3.422)	(2.417)	(3.812)	(2.669)	(3.390)	(2.555)
ISCED 4-6 (*Ref.*: ISCED 1-3)		0.156		1.396		5.333***		6.044***
		(1.445)		(1.277)		(1.550)		(1.356)
Parental HISEI		−0.874***		−1.267***		−1.067***		−0.728***
		(0.190)		(0.179)		(0.179)		(0.166)
Parental HISEI x HISEI		−0.001		0.004**		0.001		−0.002
		(0.002)		(0.002)		(0.002)		(0.002)
Basic requirements (*Ref.*: advanced)		−10.453***		−5.515***		−13.525***		−14.033***
		(1.683)		(1.162)		(1.683)		(1.413)
Pre-gymnasium		11.443***		11.713***		10.104***		10.527***
		(1.906)		(1.309)		(2.800)		(1.917)
GPA language		1.013		1.433**		1.840*		2.279*
		(0.581)		(0.526)		(0.717)		(0.915)
GPA mathematics		1.273*		1.039*		0.642		2.066**
		(0.599)		(0.470)		(0.758)		(0.706)
Constant	0.374	41.248***	−0.219	46.474***	−8.292***	48.739***	0.756	45.872***
	(0.979)	(5.263)	(0.781)	(4.645)	(1.334)	(4.545)	(1.171)	(4.239)
N	1044	1044	1233	1233	1054	1054	1084	1084
Adj. R^2	0.059	0.461	0.049	0.448	0.100	0.516	0.067	0.588

Remarks: OLS-regression, SEs in parentheses, estimates based on 25 imputed data sets using survey weights, significance levels: * $p < 0.05$, ** $p < 0.01$, *** $p < 0.001$.
Data: TREE/DAB; own calculations.

Table S5. Aspirations for social mobility

Table S6. Educational situation two years (TREE) / 15 months (DAB) after leaving compulsory education (0 = VET; 1 = academic track); Aspiration operationalised as idealistic professional aspiration of the student

	**TREE men**			**TREE women**		
	1	2	3	1	2	3
EU/neighb. countries (*Ref.*: Switz.)	−1.332	0.196	−0.033	−0.316	0.071	−0.110
Balkans, Turkey, Portugal	−0.571	2.414	2.175	−2.113	−0.514	−0.658
	(0.826)	(0.628)	(0.614)	(0.579)	(0.522)	(0.518)
Other	−1.108	−0.440	−0.131	0.472	0.133	0.029
Swiss-mixed	0.242	0.328	0.306	0.368	0.198	0.113
Born abroad (*Ref.*: Switz.)	0.706	0.319	0.310	−0.317	0.033	0.036
Parental HISEI		0.027	0.021		0.015	0.011
ISCED 4-6 (*Ref.*: ISCED 1-3)		0.708	0.668		0.496	0.459
Basic requirements (*Ref.*: adv.)		−3.477	−3.121		−2.176	−2.041
Pre-gymnasium		3.318	2.838		2.470	2.119
GPA language		0.334	0.313		0.101	0.087
GPA mathematics		0.197	0.161		0.239	0.211
Aspirations (iseiideal)			0.035			0.025
Constant	−3.351	−4.924	−6.311	−0.812	−2.242	−3.144
Observations		1044			1233	
# Bootstrap replications		481			500	

	**DAB men**			**DAB women**		
	1	2	3	1	2	3
EU15/EFTA (*Ref.*: Switz.)	1.350	1.888	1.591	0.713	1.185	1.199
Balkans, Turkey, Portugal	−0.088	1.661	0.862	−1.383	0.461	0.173
	(0.440)	(0.424)	(0.404)	(0.382)	(0.254)	(0.234)
Other	0.828	1.261	0.453	0.876	1.433	1.126
Swiss-mixed	0.255	0.766	0.405	0.032	0.351	0.217
Born abroad (*Ref.*: Switz.)	−0.388	−0.102	−0.152	−1.198	−0.772	−0.817
Parental HISEI		0.020	0.016		0.021	0.019
ISCED 4-6 (*Ref.*: 1-3)		0.517	0.195		0.486	0.260
Basic requirements (*Ref.*: adv.)		−1.803	−1.037		−4.515	−3.924
Pre-gymnasium		2.985	2.397		2.440	2.013
GPA language		0.803	0.686		0.537	0.440
GPA mathematics		0.207	0.179		0.355	0.272
Aspirations (iseiideal)			0.059			0.041
Constant	−2.770	−4.007	−6.654	−1.685	−2.786	−4.849
Observations		1054			1084	
# Bootstrap replications		498			412	

Remarks: Logit coefficient measured on the scale of the full model and selected bootstrapped SEs in parentheses. Estimates based on 25 imputed data sets using survey weights. *Data:* TREE/DAB; own calculations.

Table S7. Selected indirect effects and percentage mediated of migration background on upper secondary track attended in Table S6

	TREE		DAB	
	Men	Women	Men	Women
Indirect effects				
M2 ⇒ M3	0.231 (0.102)	–	0.772 (0.158)	–
Percent mediated				
M2 ⇒ M3	9.7% (10.6)	–	47.3% (12.6)	–
# Bootstrap replications	491	–	496	–

Remarks: Logit coefficient, percentage mediated and bootstrapped SEs in parentheses.
Estimates based on coefficients reported in Table S6.

Table S8. Educational situation two years (TREE) / 15 months (DAB) after leaving compulsory education (0 = VET; 1 = academic track); Results without stratification by gender

	TREE			DAB		
	1	2	3	1	2	3
EU/neighb. countries (*Ref.*: Switz.)	−0.717	0.118	−0.075	0.993	1.430	1.393
Balkans, Turkey, Portugal	−1.220	0.828	0.655	−2.113	−0.514	−0.658
	(0.497)	(0.458)	(0.444)	(0.324)	(0.234)	(0.223)
Other	−0.206	0.032	−0.044	0.856	1.300	0.892
Swiss-mixed	0.289	0.224	0.157	0.171	0.562	0.355
Born abroad (*Ref.*: Switz.)	0.290	0.262	0.251	−0.755	−0.441	−0.530
Women (*Ref.*: men)	1.764	1.230	1.293	1.226	0.603	0.475
Parental HISEI		0.019	0.041		0.021	0.062
ISCED 4-6 (*Ref.*: ISCED 1-3)		0.514	0.480		0.471	0.212
Basic requirements (*Ref.*: adv.)		−2.546	−2.311		−2.548	−1.961
Pre-gymnasium		2.763	2.403		2.635	2.174
GPA language		0.187	0.171		0.619	0.537
GPA mathematics		0.210	0.177		0.302	0.240
Aspirations (iseiideal)			0.026			0.044
Constant	−2.689	−3.879	−4.907	−0.812	−3.618	−5.635
Observations		2277			2138	
# Bootstrap replications		493			500	

Remarks: Logit coefficient measured on the scale of the full model and selected bootstrapped SEs in parentheses.
Estimates based on 25 imputed data sets using survey weights.
Data: TREE/DAB; own calculations.

Table S9. Educational situation 15 months (DAB) after leaving compulsory education (0 = VET; 1 = academic track); information on aspirations and school type attended at the end of Grade 9

	DAB men			DAB women		
	1	2	3	1	2	3
EU15/EFTA (*Ref.*: Switz.)	1.746	1.841	1.225	1.133	1.126	0.861
Balkans, Turkey, Portugal	0.184	1.778	1.053	−0.958	0.446	0.089
	(0.485)	(0.425)	(0.384)	(0.487)	(0.320)	(0.309)
Other	1.076	1.525	0.782	1.190	1.451	0.981
Swiss-mixed	0.253	0.436	0.029	0.157	0.316	0.072
Born abroad (*Ref.*: Switz.)	−0.079	−0.114	−0.098	−1.118	−0.546	−0.741
Parental HISEI		0.027	0.082		0.023	0.069
ISCED 4-6 (*Ref.*: 1-3)		0.346	0.160		0.531	0.303
Basic requirements (*Ref.*: adv.)		−1.550	−0.862		−2.717	−2.048
Pre-gymnasium		3.997	3.082		3.351	2.568
GPA language		−0.029	−0.060		−0.149	−0.170
GPA mathematics		0.103	0.068		0.314	0.227
Aspirations (dfiseit3)			0.063			0.051
Constant	−3.032	−5.053	−7.686	−1.555	−3.373	−5.841
Observations		1030			1060	
# Bootstrap replications		498			489	

Remarks: Logit coefficient measured on the scale of the full model and selected bootstrapped SEs in parentheses. Estimates based on 25 imputed data sets using survey weights. *Data:* TREE/DAB; own calculations.

Table S10. Selected indirect effects and percentage mediated of migration background on upper secondary track attended in Table S9

	TREE		DAB	
	Men	Women	Men	Women
Indirect effects				
M2 $\Rightarrow$ M3	–		0.676 (0.140)	–
Percent mediated				
M2 $\Rightarrow$ M3	–		39.6% (23.6)	–
# Bootstrap replications	–		493	–

Remarks: Logit coefficient, percentage mediated and bootstrapped SEs in parentheses.
Estimates based on coefficients reported in Table S9.

REFERENCES

Dollmann, J. (2017). Positive choices for all? SES- and gender-specific premia of immigrants at educational transitions. *Research in Social Stratification and Mobility* 49, 20–31. doi:10.1016/j.rssm.2017.03.001

Kao, G. and Tienda, M. (1995). Optimism and achievement: The educational performance of immigrant youth. *Social Science Quarterly* 76, 1–19
