# Supplementary material for: Gendered ethnic choice effects at the transition to upper secondary education in Switzerland
Source: Front Sociol. 2023 Apr 17;8:1158071. doi: 10.3389/fsoc.2023.1158071 (PMC10150114; doi:10.3389/fsoc.2023.1158071)
Supplement: Supplementary file 2 [file Data_Sheet_2.pdf]

# Appendix

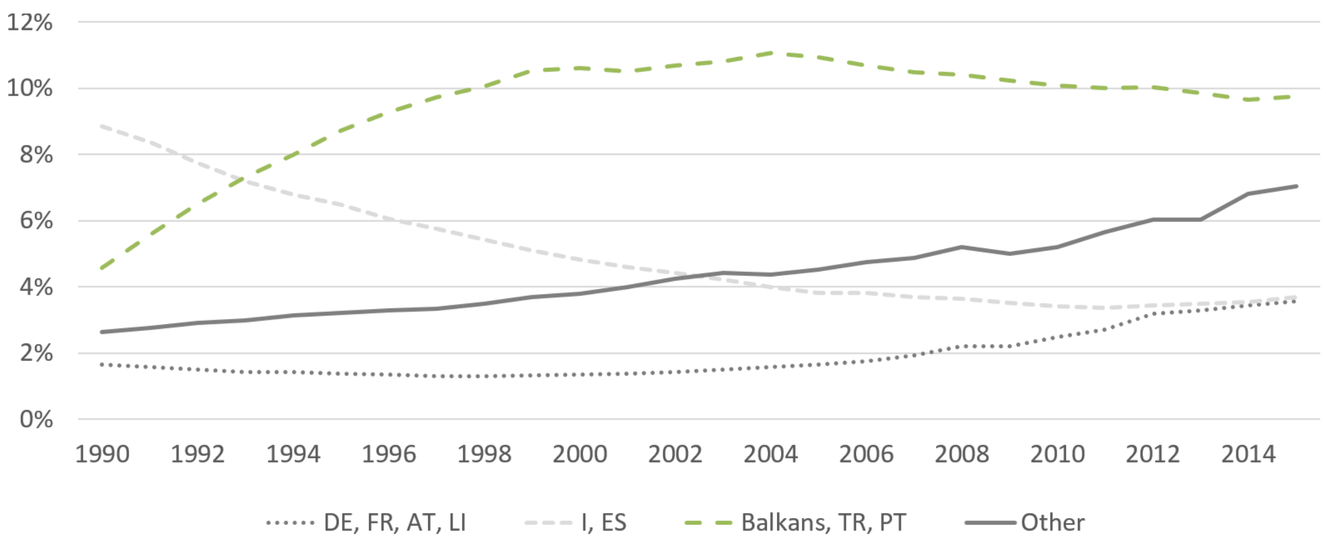


*Remarks:* Data requested from Federal Statistical Office 01/2018; own calculations.

**Figure 2.** Change in the proportion of students with a migrant background (based on student's nationality) at lower secondary level, 1990 – 2015

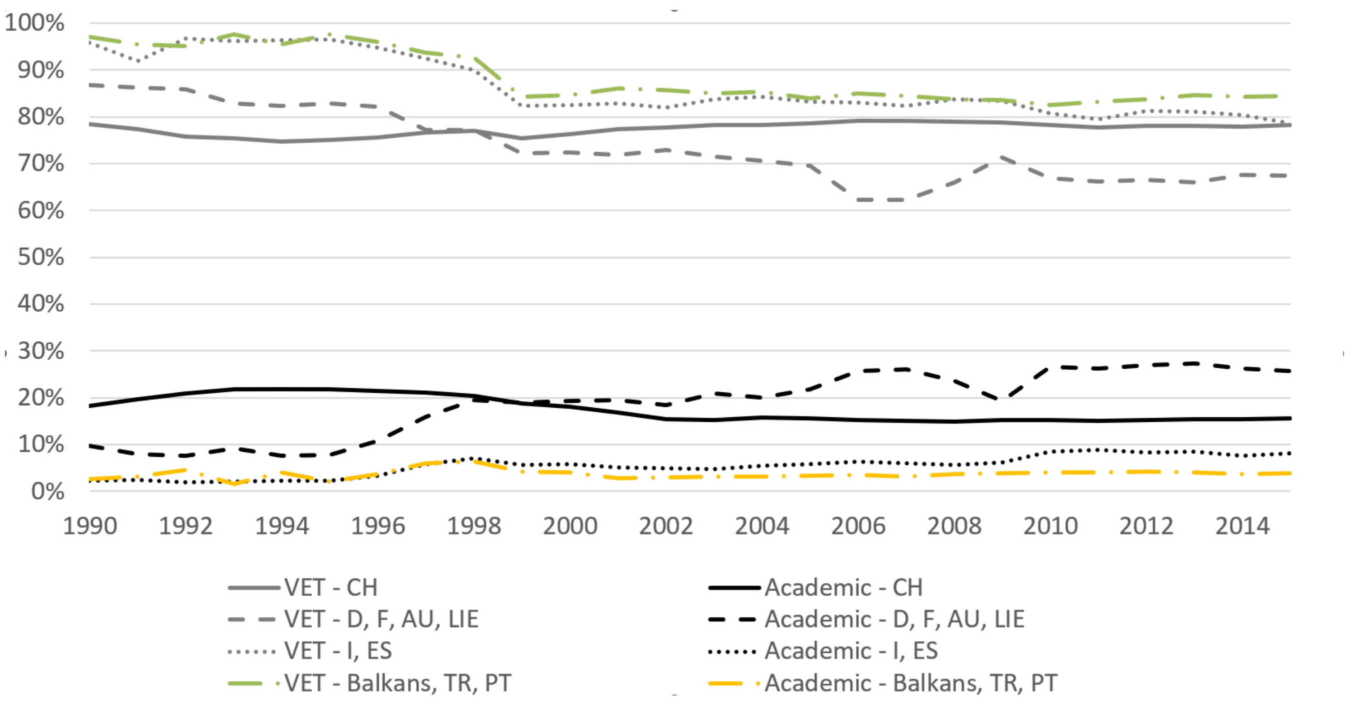


*Remarks:* Data requested from Federal Statistical Office 02/2020; own calculations.

**Figure 3.** Upper secondary track by migration background (based on student's nationality), men: 1990 – 2015, German-speaking Switzerland

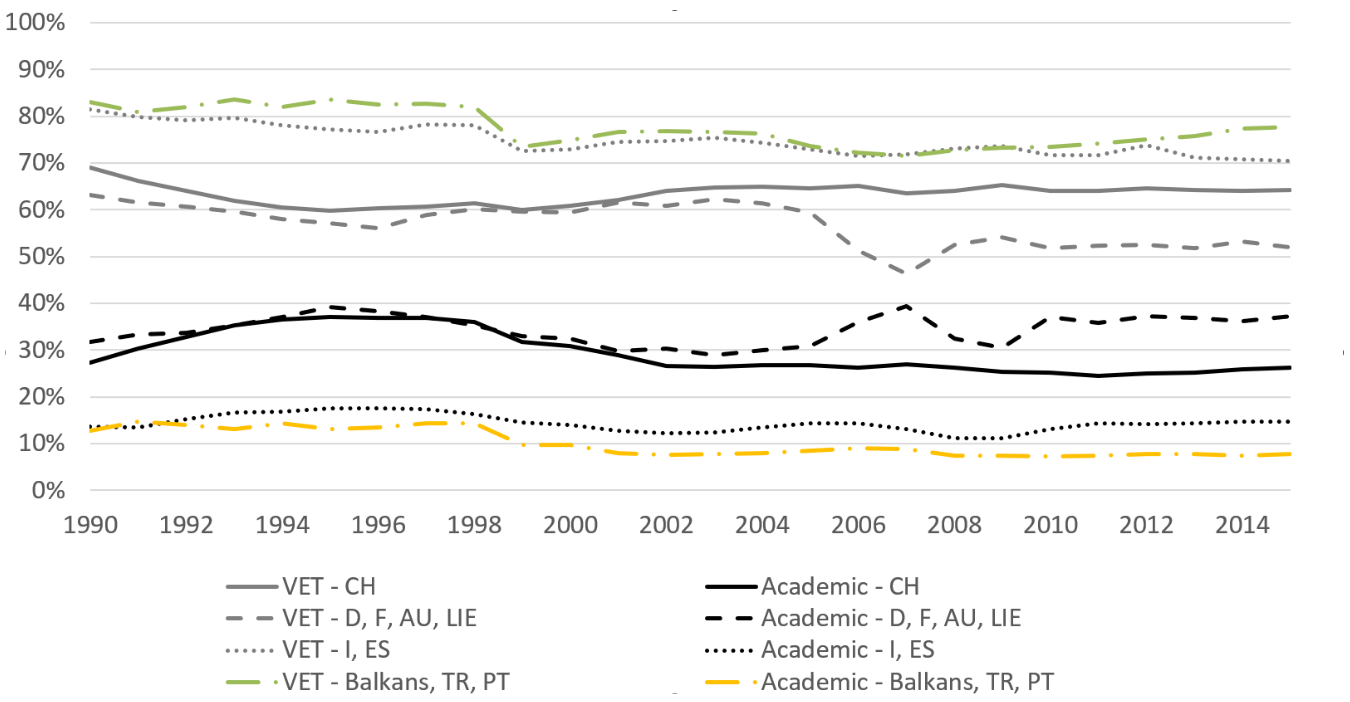


*Remarks:* Data requested from Federal Statistical Office 02/2020; own calculations.

**Figure 4.** Upper secondary track by migration background (based on student's nationality), women: 1990 – 2015, German-speaking Switzerland

| | **Men** ($N = 1044$) | | **Women** ($N = 1233$) | |
|---|---|---|---|---|
| | prop./mean | 95%CI/SD | prop./mean | 95%CI/SD |
| Upper secondary track | | | | |
| VET | 0.8140 | 0.7601 0.8679 | 0.6339 | 0.5728 0.6950 |
| Academic track | 0.1860 | 0.1321 0.2399 | 0.3661 | 0.3050 0.4272 |
| Country of birth parents | | | | |
| Switzerland | 0.7100 | 0.6664 0.7537 | 0.7345 | 0.6963 0.7726 |
| EU/neighbours | 0.0428 | 0.0235 0.0620 | 0.0423 | 0.0258 0.0587 |
| Balkans, Turkey, Portugal | 0.1197 | 0.0872 0.1521 | 0.0723 | 0.0486 0.0960 |
| Other countries | 0.0285 | 0.0131 0.0439 | 0.0254 | 0.0128 0.0379 |
| Switzerland-mixed | 0.0990 | 0.0722 0.1258 | 0.1256 | 0.1014 0.1498 |
| Country of birth child | | | | |
| Switzerland | 0.8861 | 0.8550 0.9173 | 0.9181 | 0.8931 0.9431 |
| Born abroad | 0.1139 | 0.0827 0.1450 | 0.0819 | 0.0569 0.1069 |
| Highest educational level parents | | | | |
| ISCED 1-3 | 0.3097 | 0.2592 0.3603 | 0.3174 | 0.2806 0.3543 |
| ISCED 4-6 | 0.6903 | 0.6397 0.7408 | 0.6826 | 0.6457 0.7194 |
| Parental HISEI | 47.1318 | 16.450 | 48.3062 | 15.964 |
| School type attended in Grade 9 | | | | |
| Basic requirements | 0.3814 | 0.2979 0.4649 | 0.2387 | 0.1744 0.3030 |
| Advanced requirements | 0.3797 | 0.3000 0.4594 | 0.4501 | 0.3711 0.5290 |
| Pre-gymnasium | 0.2389 | 0.1661 0.3118 | 0.3112 | 0.2317 0.3907 |
| GPA language ($z$-stand.) | −0.2393 | 1.073 | 0.1665 | 1.013 |
| GPA mathematics ($z$-stand.) | 0.0789 | 0.999 | −0.0536 | 0.978 |
| Aspiration for social mobility | 1.9079 | 19.625 | 1.2507 | 19.197 |

*Remarks*: Estimates based on 25 imputed data sets using survey weights. Number of imputed missings on the overall sample size $N = 2277$: Country of birth parents (9), Country of birth child (8), Highest educational level parents (50), Parental HISEI (32), GPA language (89), GPA mathematics (87), Aspiration for social mobility (684). *Data:* TREE; own calculations.

**Table 3.** Descriptives of sample used in Table 1: TREE sample

| | **Men** ($N = 1054$) | | **Women** ($N = 1084$) | |
|---|---|---|---|---|
| | prop./mean | 95%CI/SD | prop./mean | 95%CI/SD |
| Upper secondary track | | | | |
| VET | 0.8134 | 0.7572 0.8695 | 0.6866 | 0.6195 0.7537 |
| Academic track | 0.1866 | 0.1305 0.2428 | 0.3134 | 0.2463 0.3805 |
| Country of birth parents | | | | |
| Switzerland | 0.5886 | 0.5429 0.6344 | 0.5714 | 0.5164 0.6263 |
| EU-15/EFTA | 0.0325 | 0.0198 0.0452 | 0.0472 | 0.0316 0.0627 |
| Balkans, Turkey, Portugal | 0.1323 | 0.1016 0.1630 | 0.1502 | 0.1127 0.1876 |
| Other countries | 0.0922 | 0.0685 0.1159 | 0.0789 | 0.0534 0.1040 |
| Switzerland-mixed | 0.1544 | 0.1237 0.1851 | 0.1524 | 0.1243 0.1804 |
| Country of birth child | | | | |
| Switzerland | 0.9148 | 0.8925 0.9371 | 0.9142 | 0.8926 0.9358 |
| Born abroad | 0.0852 | 0.0629 0.1075 | 0.0858 | 0.0642 0.1074 |
| Highest educational level parents | | | | |
| ISCED 1-3 | 0.6585 | 0.6169 0.7002 | 0.6952 | 0.6586 0.7317 |
| ISCED 4-6 | 0.3415 | 0.2998 0.3831 | 0.3048 | 0.2683 0.3414 |
| Parental HISEI | 53.9295 | 21.090 | 52.6233 | 21.154 |
| School type attended in Grade 8 | | | | |
| Basic requirements | 0.3611 | 0.2855 0.4366 | 0.2644 | 0.1995 0.3294 |
| Advanced requirements | 0.5259 | 0.4452 0.6065 | 0.5589 | 0.4740 0.6438 |
| Pre-gymnasium | 0.1130 | 0.0489 0.1771 | 0.1767 | 0.0925 0.2609 |
| GPA language ($z$-stand.) | −0.1406 | 0.998 | 0.2988 | 0.913 |
| GPA mathematics ($z$-stand.) | 0.1581 | 0.953 | −0.0106 | 0.978 |
| Aspiration for social mobility | −3.0496 | 27.333 | 3.3545 | 26.099 |

*Remarks*: Estimates based on 25 imputed data sets using survey weights. Number of imputed missings on the overall sample size $N = 2138$: Country of birth parents (124), Country of birth child (163), Highest educational level parents (148), Parental HISEI (185), GPA language (188), GPA mathematics (223), Aspiration for social mobility (269). *Data:* DAB panel study; own calculations.

**Table 4.** Descriptives of sample used in Table 1: DAB sample
